# Expatriates ill after travel: Results from the Geosentinel Surveillance Network

**DOI:** 10.1186/1471-2334-12-386

**Published:** 2012-12-31

**Authors:** Poh-Lian Lim, Pauline Han, Lin H Chen, Susan MacDonald, Prativa Pandey, DeVon Hale, Patricia Schlagenhauf, Louis Loutan, Annelies Wilder-Smith, Xiaohong M Davis, David O Freedman

**Affiliations:** 1 Department of Infectious Diseases, Institute of Infectious Disease & Epidemiology, Tan Tock Seng Hospital, Singapore, Singapore; 2 Centers for Disease Control and Prevention, Atlanta, Georgia, USA; 3 Mount Auburn Hospital, Cambridge, Massachusetts, USA; 4 Harvard Medical School, Boston, Massachusetts, USA; 5 University Hospital of Northern British Columbia, Prince George, Canada; 6 CIWEC Clinic Travel Medicine Center, Katmandu, Nepal; 7 Division of Infectious Diseases, University of Utah School of Medicine, Salt Lake City, Utah, USA; 8 University of Zurich Centre for Travel Medicine, WHO Collaborating Centre for Travellers’ Health, University of Zurich, Zurich, Switzerland; 9 University of Geneva, Geneva, Switzerland; 10 Institute of Public Health, University of Heidelberg, Heidelberg, Germany; 11 Division of Infectious Diseases, University of Alabama at Birmingham, Birmingham, Alabama, USA

**Keywords:** Expatriate, Travelers, GeoSentinel, Malaria

## Abstract

**Background:**

Expatriates are a distinct population at unique risk for health problems related to their travel exposure.

**Methods:**

We analyzed GeoSentinel data comparing ill returned expatriates with other travelers for demographics, travel characteristics, and proportionate morbidity (PM) for travel-related illness.

**Results:**

Our study included 2,883 expatriates and 11,910 non-expatriates who visited GeoSentinel clinics ill after travel. Expatriates were more likely to be male, do volunteer work, be long-stay travelers (>6 months), and have sought pre-travel advice. Compared to non-expatriates, expatriates returning from Africa had higher proportionate morbidity (PM) for malaria, filariasis, schistosomiasis, and hepatitis E; expatriates from the Asia-Pacific region had higher PM for strongyloidiasis, depression, and anxiety; expatriates returning from Latin America had higher PM for mononucleosis and ingestion-related infections (giardiasis, brucellosis). Expatriates returning from all three regions had higher PM for latent TB, amebiasis, and gastrointestinal infections (other than acute diarrhea) compared to non-expatriates. When the data were stratified by travel reason, business expatriates had higher PM for febrile systemic illness (malaria and dengue) and vaccine-preventable infections (hepatitis A), and volunteer expatriates had higher PM for parasitic infections. Expatriates overall had higher adjusted odds ratios for latent TB and lower odds ratios for acute diarrhea and dermatologic illness.

**Conclusions:**

Ill returned expatriates differ from other travelers in travel characteristics and proportionate morbidity for specific diseases, based on the region of exposure and travel reason. They are more likely to present with more serious illness.

## Background

Expatriates are a diverse group who must adapt to their host culture, who have a longer duration of exposure to country-related hazards, and who have the opportunity to modify risks in their immediate environment [1]. With increasing globalization, expatriate travel has grown substantially, along with an increasing need to understand the health issues for these travelers. Despite this, there is as yet no definitive answer to the number of expatriates worldwide. United Nations statistics in 2005 indicated 32 million expatriates in the 34 countries of the Organization for Economic Cooperation and Development (OECD) [2]. Another study estimated 3 million expatriates in the United Arab Emirates alone [3]. Given all the countries not included in the estimates above, the total number of expatriates worldwide may number well over 40–50 million.

Expatriates have been defined by Foyle as those who take up residence in another country for occupational purposes, returning to their country of origin when their assignment is completed [4]. Using local infrastructure and longer-term residence abroad expose expatriates to health risks. Expatriates are therefore considered distinct from tourists, who use commercial lodgings such as hotels or hostels, or travelers visiting friends and relatives (VFRs), who stay in local homes as guests. Expatriates are also distinct from immigrants in their nonpermanent residence in their destination country, although many expatriates reside abroad for long periods, from months to years.

Few systematic studies have examined the full spectrum of health problems among different expatriate subgroups and their destinations. We present data on ill returned expatriates seen after travel at GeoSentinel sites worldwide to better guide clinicians and organizations responsible for expatriate health.

## Methods

### Data collection

The GeoSentinel Surveillance Network consists of specialized travel or tropical medicine clinics on 6 continents contributing data on travelers seen during or after travel. Sentinel surveillance data, including demographic and travel characteristics, are collected by using standard, anonymous reporting forms, as described [5]. Final diagnoses were selected from a standardized list of over 500 diagnostic codes, based on the best available reference diagnostics in the site country. These diagnoses were grouped into 21 broad syndrome categories. Individual patients could have more than one final diagnosis.

### Definitions

Site clinicians marked a traveler as “expatriate”, according to the GeoSentinel data entry definition as those living in a destination country with an independent residence, using mostly the infrastructure used by local residents of the same economic class, independent of duration of residence. “Non-expatriates” were defined as those who were not marked to be an “expatriate”, including business and volunteer travelers staying in hostels and hotels. An expatriate was further classified by their travel reason (e.g., business, volunteer). Countries of exposure were categorized into one of 4 regions: Africa, Asia-Pacific, Latin America, and “Other” (Europe, North America, Middle East). Five syndromic groupings of diagnoses were analyzed: acute diarrhea, systemic febrile illness, other gastrointestinal (GI) disorders, dermatologic, and respiratory illness. We further categorized individual diagnostic codes by mode of transmission: vector-borne, ingestion, respiratory, blood/body fluid/sexual, environmental contact and noninfectious, similar to previous studies [6]. Data were analyzed for patients evaluated from 18 March 1997 to 31 May 2011. Patients were included who presented ill to a GeoSentinel clinic after either expatriate or non-expatriate travel. Immigrants and VFRs were excluded. Also excluded were those whose reason for travel was tourism, education, military, or medical tourism, as none of these records were classified as an expatriate. Only patients whose reason for travel was business or missionary/volunteer/aid work (abbreviated as volunteer hereafter) were included.

### Ethics

The GeoSentinel data-collection protocol was reviewed by the institutional review board officer at the National Center for Infectious Diseases at the Centers for Disease Control and Prevention and classified as public health surveillance and not as human-subjects research requiring submission to institutional review boards.

### Statistical analysis

Demographic and travel characteristics of expatriates were compared to non-expatriates, as well as for syndromic illness and specific diagnostic groupings. Proportionate morbidity (PM) was expressed as the number of patients with a specific syndrome or diagnosis per 1,000 ill returned expatriates and non-expatriates. A sub-analysis of expatriates based on travel reason (business vs. volunteer) was also performed on syndromic illness and specific diagnostic groupings. Categorical variables were compared by using the chi-square test and analyzed by using bivariate odds ratios and 95% confidence intervals. For select diagnostic groupings, multivariable logistic regression was used, adjusting for factors found to be significant in bivariate analyses. SAS 9.2 was used in all statistical analyses. A value of <0.05 was considered statistically significant.

## Results

**Figure 1:**
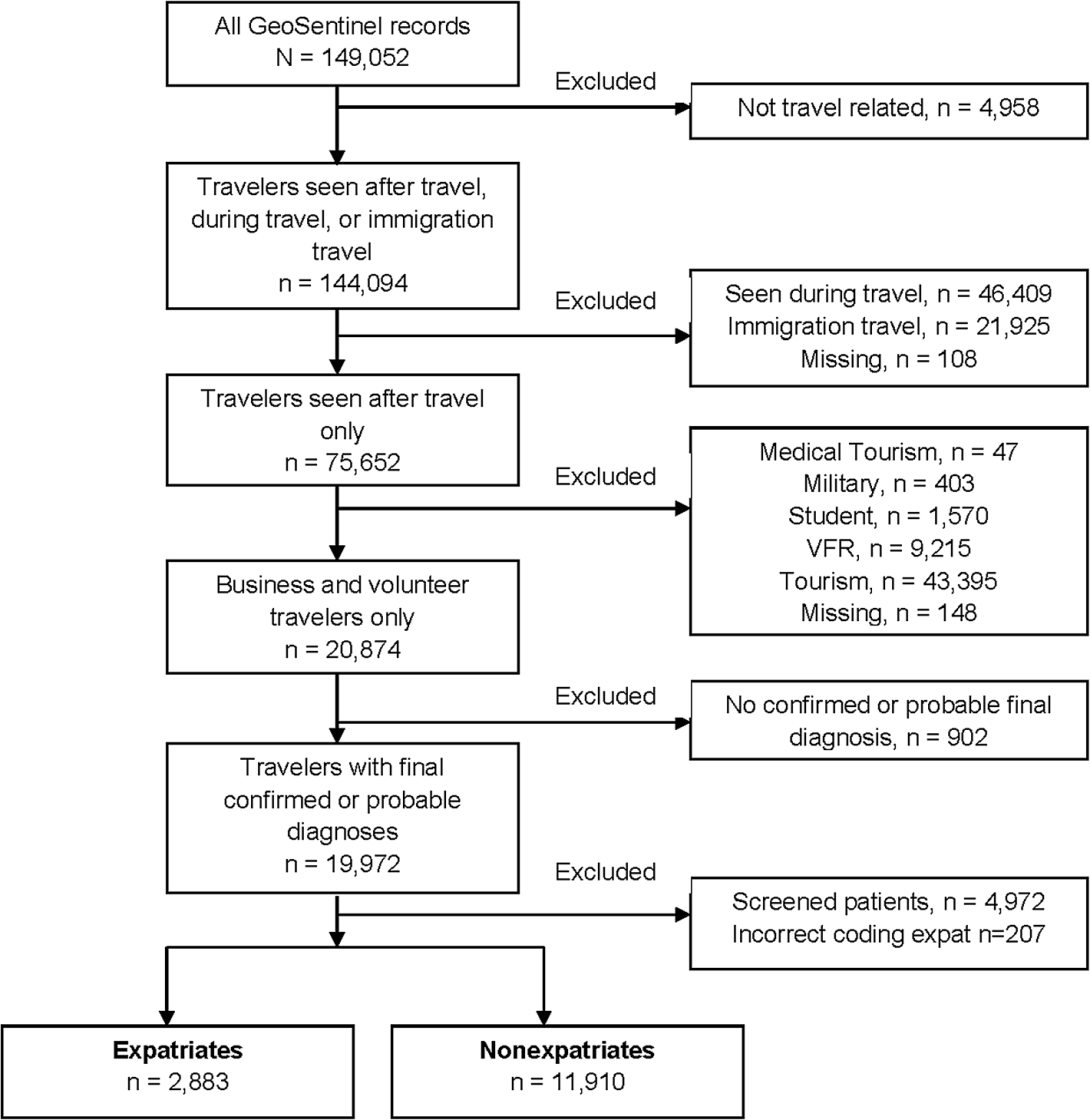
Flow chart for analysis of expatriate travelers seen in GeoSentinel Clinics, March 1997 – May 2011.

From March 1997 to May 2011, 14,793 business and volunteer travelers visited GeoSentinel sites after travel with confirmed or probable travel-related final diagnoses; 2,883 (19.5%) were ill returned expatriates (Figure 1). Expatriates were more likely than other travelers to be male, to have longer trip durations, to travel for volunteer work, and to have sought pre-travel advice (Table 1).

**Table 1:** Characteristics of ill returned travelers seen at GeoSentinel Clinics, expatriates versus non-expatriates, March 1997 – May 2011

	**All**		
	**TOTAL**	**Expatriates**	**Non-expatriates**
	**(N=14,793)**	**(n=2,883)**	**(n=11,910)**
	**Percentage, %**		
Male^*^	58	64	56
Age^*^			
<=19	4	6	4
20-64	92	91	93
65+	4	4	3
Sought pre-travel advice^*^	60	70	58
Travel reason^*^			
Business	58	41	62
Volunteer	42	59	38
Trip duration^*^			
<1 month	44	11	53
1-6 months	25	20	26
>6 months	18	51	10
Unknown	13	18	11
Exposure region^*^			
Africa	38	39	37
Asia-Pacific	28	23	30
Latin America	19	25	17
Other ^a^	6	7	6
Unknown	9	6	9
**Patient type**			
Inpatient	10	11	10
Outpatient	90	89	90

^*^χ^*2*^*p-value < 0.01, comparing expatriates with non-expatriates.*

^a^ Other includes Europe, Middle East, and North America and does not include unknown region (n=1277).

Comparing expatriates with non-expatriates for syndromic groupings of diagnoses, expatriates ill after travel had lower PM for acute diarrhea and dermatologic and respiratory illnesses but higher PM for other GI disorders (Table 2).

**Table 2:** Comparison of proportionate morbidity per 1,000 ill returned travelers seen at GeoSentinel clinics by syndrome and diagnostic groupings, A) expatriate versus nonexpatriate; B) business and volunteer expatriates versus business and volunteer non-expatriates, March 1997 – May 2011

		** ALL**		**Business travelers**			** Volunteer travelers**		
	**Expatriate**	**Non-expatriate**	**p-value (bivariate)**	**Expatriate**	**Non-expatriate**	**p-value (bivariate)**	**Expatriate**	**Non-expatriate**	**p-value (bivariate)**
	**PM per 1,000**			**PM per 1,000**			**PM per 1,000**		
Acute Diarrhea	136	233	<0.01	131	231	<0.01	139	236	<0.01
Febrile/Systemic Illness	195	201	0.46	265	212	<0.01	147	183	<0.01
Dermatologic	88	121	<0.01	103	115	0.22	79	130	<0.01
GI, Other	121	74	<0.01	112	71	<0.01	127	79	<0.01
Respiratory	49	86	<0.01	75	102	<0.01	31	59	<0.01
***Vector-borne***									
Malaria	84	62	<0.01	109	62	<0.01	67	61	0.37
Dengue	21	23	0.44	31	22	0.05	13	25	<0.01
Leishmaniasis	5	5	0.73	2	4	0.42	8	6	0.60
Rickettsiosis	3	4	0.56	6	5	0.64	1	2	0.41
Filariasis	14	4	<0.01	3	2	0.48	21	6	<0.01
***Ingestion***									
Typhoid & Paratyphoid	4	5	0.83	6	3	0.18	3	6	0.17
Hepatitis A	3	2	0.35	7	3	0.03	0.6	2	0.69
Hepatitis E	3	0.4	<0.01	3	0.5	0.06	4	0.2	<0.01
GI disease (parasitic)	15	13	0.28	18	11	0.05	14	16	0.64
GI disease (bacterial)	11	27	<0.01	14	30	<0.01	9	23	<0.01
Amebiasis	32	17	<0.01	19	16	0.32	41	19	<0.01
Giardiasis	26	32	0.10	24	28	0.36	28	39	0.05
Brucellosis	2	0.4	<0.01	0.8	0.5	0.52	4	0.2	<0.01
***Respiratory Contact***									
Influenza	7	17	<0.01	13	23	0.03	3	9	0.01
Latent TB	19	8	<0.01	26	9	<0.01	14	5	<0.01
Active TB	4	2	0.02	6	2	0.03	3	1	0.07
***Blood/Body Fluid/Sexual***									
HIV	7	2	<0.01	13	3	<0.01	2	1	0.27
Hepatitis B	1	1	0.91	0.8	1	1.00	0.6	0	0.27
IMS^1^ – EBV^2^/CMV^3^	16	7	<0.01	8	8	0.89	22	5	<0.01
***Environmental Contact***									
Schistosomiasis	15	9	<0.01	10	5	0.02	19	15	0.26
Strongyloidiasis	14	5	<0.01	7	3	0.03	19	9	<0.01
Ectoparasites	4	9	<0.01	5	7	0.42	4	12	<0.01
Animal Bites	3	3	0.99	6	2	0.03	0.6	4	0.05
***Non-infectious***									
Fracture & Dislocation	1	0.4	0.20	0.8	0.3	0.33	1	0.7	0.62
Depression	8	3	<0.01	9	2	<0.01	7	5	0.24
Anxiety & Stress	7	3	<0.01	3	4	1.00	10	3	<0.01
Cardiac Disease	2	1	0.04	4	1	0.02	0.6	0.2	0.47

^1^IMS=infectious mononucleosis, ^2^EBV=Epstein-Barr virus; ^3^CMV=Cytomegalovirus.

Region of exposure was associated with differences in proportionate morbidity when comparing expatriates and non-expatriates after travel (Table 3). For exposure in Africa, expatriates had significantly higher PM compared with non-expatriates for infections transmitted by mosquitoes (malaria, filariasis), by environmental contact (schistosomiasis), and by ingestion (amebiasis, hepatitis E). For exposure in the Asia-Pacific region, expatriates had a higher PM than non-expatriates for strongyloidiasis, filariasis, depression, and anxiety/stress. Compared with the African region, malaria in the Asia-Pacific region was predominantly due to non-falciparum species. By contrast, expatriates exposed in Latin America had higher PM than non-expatriates for infectious mononucleosis and ingestion-related infections (amebiasis, giardiasis, and brucellosis). Across all three regions (Africa, Asia-Pacific, and Latin America), expatriates had higher PM for latent TB and amebiasis.

**Table 3:** Proportionate morbidity per 1,000 ill returned travelers seen at GeoSentinel clinics, for syndrome and diagnostic groupings by region, expatriates versus non-expatriates, March 1997 – May 2011

	** AFRICA (n=5575)**		**ASIA-PACIFIC (n=4200)**		**LATIN AMERICA (n=2787)**		**OTHER REGIONS**^**¥**^**(n=954)**		p-value *
	**Expatriate**	**Non-expatriate**	**Expatriate**	**Non-expatriate**	**Expatriate**	**Non-expatriate**	**Expatriate**	**Non-expatriate**	
**PM per 1,000**									
**Syndrome Groupings**									
Acute Diarrhea	139	226	147	273	129	240	113	171	a,b,c
Febrile/Systemic Illness	289	263	195	203	95	148	77	103	c
Dermatologic	73	111	94	111	103	179	93	122	a,c
GI, Other	101	65	140	68	136	70	124	86	a,b,c
Respiratory	45	67	68	104	23	58	88	186	a,b,c,d
**Diagnostic Groupings**									
***Vector-borne***									
Malaria	178	121	51	43	7	16	0	3	a
*P.falciparum*	115	86	9	13	1	4	0	0	a
*Malaria, other*^*£*^	56	30	40	31	4	12	0	3	a
Dengue	6	7	52	46	20	36	0	0	
Leishmaniasis	3	3	2	1	15	15	0	12	
Rickettsiosis	4	8	5	1	0	2	0	1	
Filariasis	31	7	6	1	3	3	0	1	a,b
***Ingestion***									
Typhoid & Paratyphoid	4	3	10	10	1	3	0	0	
Hepatitis A	3	2	6	2	1	1	5	4	
Hepatitis E	5	0.2	3	1	1	0	0	0	a
GI (parasitic)	14	8	21	13	14	23	10	8	
GI (bacterial)	9	27	13	39	12	18	5	29	a,b
Amebiasis	31	16	30	16	42	22	21	17	a,b,c
Giardiasis	33	32	30	37	14	30	10	21	c
Brucellosis	2	0.2	2	1	4	0	5	3	c
***Respiratory Contact***									
Influenza	6	14	9	22	3	7	21	45	b
Latent TB	13	4	25	7	15	5	36	21	a,b,c
Active TB	3	1	3	2	5	2	10	4	
***Blood/Body Fluid/Sexual***									
HIV	5	2	5	2	3	3	41	5	d
Hepatitis B	2	0	0	1	0	0	0	1	
IMS – EBV/CMV	5	5	15	9	34	8	26	9	c
***Environmental Contact***									
Schistosomiasis	36	19	1	2	1	2	5	5	a
Strongyloidiasis	13	6	16	3	15	6	10	3	b
Ectoparasites	5	10	0	4	7	20	0	7	c
Animal Bites	2	3	7	4	0	3	5	3	
***Non-infectious***									
Fracture & Dislocation	0	0.4	2	1	0	1	10	0	d
Depression	3	3	12	2	8	3	16	7	b
Anxiety & Stress	8	4	7	1	4	3	21	5	b
Cardiac Disease	3	0.4	2	0.6	0	1	0	1	

* Comparing expatriate to non-expatriate, if p-value < 0.01, denoted a for Africa, b - Asia-Pacific, c - Latin America, d - Other.

^¥^ Other regions include Europe, Middle East, and North America and does not include unknown region (n=1277).

^£^ Malaria, other includes species unknown, *P. vivax*, *P. malariae*, *P. ovale*, P.v., and mefloquine-resistant.

When expatriates were grouped by travel reason, business expatriates had higher PM for certain febrile systemic illnesses (malaria and dengue), vaccine-preventable infections (hepatitis A), HIV, and animal bites compared to business non-expatriates (Table 2). In contrast, volunteer expatriates had higher PM for anxiety/stress, infectious mononucleosis, and parasitic infections (filariasis, amebiasis, and strongyloidiasis) compared to volunteer non-expatriates.

**Figure 2:**
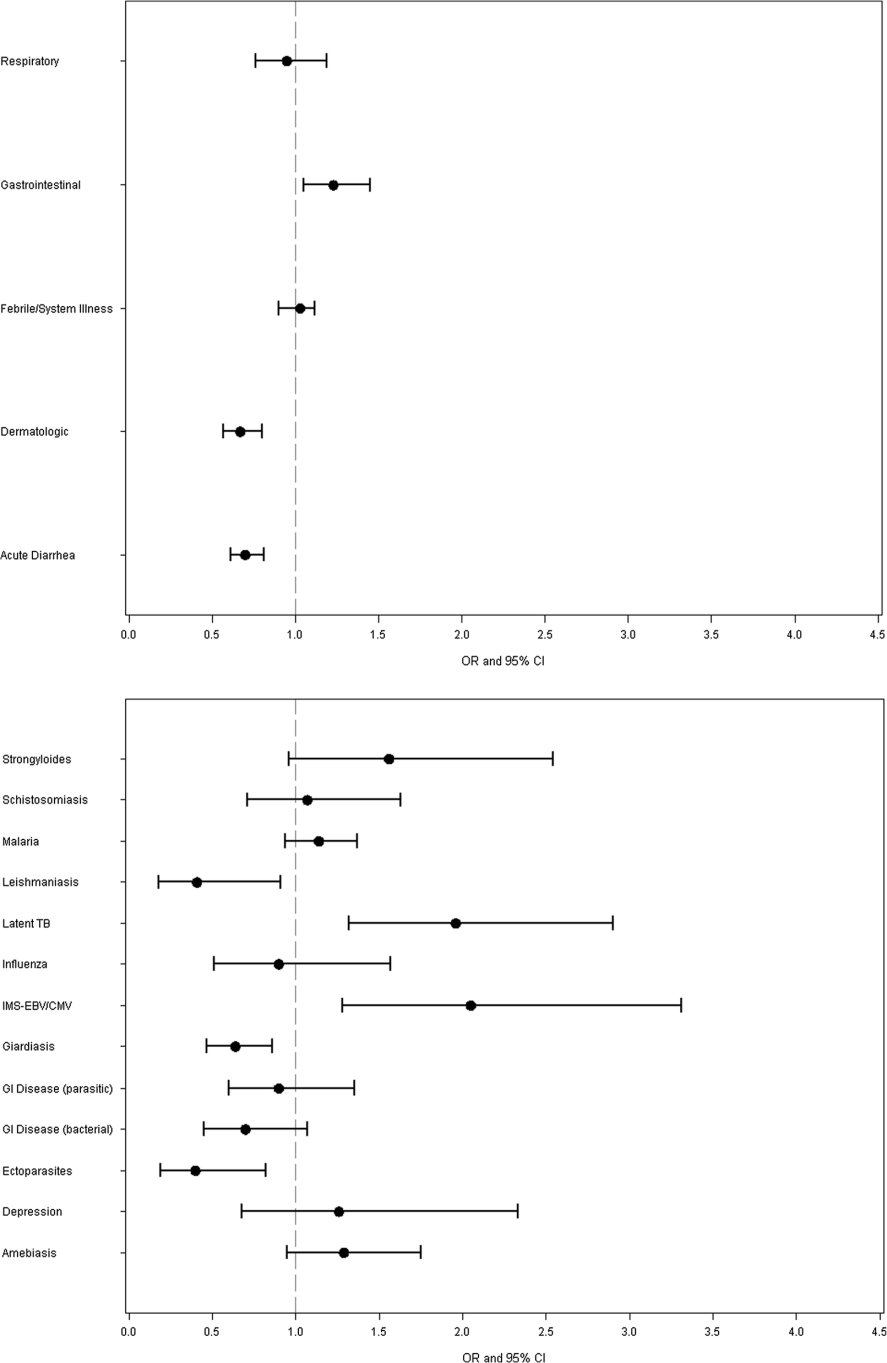
Adjusted odds ratios by major syndrome groupings and selected diagnostic groupings of ill returned travelers seen at GeoSentinel clinics, expatriates vs. non-expatriates, March 1997 – May 2011 * Adjusted for sex, age, travel reason, trip duration, and exposure region.

After the data were adjusted for sex, age, travel reason, trip duration, and exposure region, expatriates had higher adjusted odds ratios than non-expatriates for gastrointestinal disorders other than acute diarrhea, latent TB and infectious mononucleosis syndrome (IMS) and lower odds ratios for acute diarrhea and dermatologic illness, as well as leishmaniasis, giardiasis, and ectoparasites (Figure 2).

## Discussion

This analysis compares 2,883 ill returned expatriates with 11,910 ill non-expatriates to elucidate differences in travel patterns and illness seen after travel. Our expatriate definition was formulated independent of travel duration; therefore, there could have been situations where expatriates returned ill to their country of citizenship after only a short duration of travel. We have not excluded these cases from analysis because early repatriation did not alter the fact that they had resided abroad as expatriates.

Destination remains one of the most important factors influencing travel-related illness and is a major focus for both travel medicine and occupational health clinicians. Our analysis, showing higher PM for both falciparum and non-falciparum malaria among expatriates exposed in Africa and the Asia-Pacific region, respectively, should guide pre-travel advice and post-travel assessment for these travelers. The highest PM for filariasis and schistosomiasis was observed among expatriates exposed in Africa, indicating the importance of focusing preventive advice on mosquito-bite precautions and environmental contact for these expatriates. Asia-bound expatriates, on the other hand, may need to be advised about reducing exposure to tuberculosis, non-falciparum malaria, and strongyloidiasis. Expatriates exposed in Latin America, with the highest observed PM among all regions for amebiasis and infectious mononucleosis, may need to be advised about exposure to these illnesses.

Malaria carried the highest PM of all specific diagnoses, underscoring its importance among expatriates returning with symptomatic illness. This finding was true both for expatriates returning from Africa and the Asia-Pacific region. Although our study cannot estimate malaria risk, this finding is consistent with past studies that documented high rates of malaria in a wide range of expatriates, including missionaries, embassy personnel, urban expatriates, and Peace Corps volunteers in rural settings [7-10].

Our study does not provide any information about malaria chemoprophylaxis. Although two-thirds of our expatriate sample had sought pre-travel advice, compliance with chemoprophylaxis among expatriates is known to be suboptimal, possibly related to their longer duration of travel. One study in Zaire showed the use of malaria prophylaxis in missionaries at risk has ranged from 19% to 62% [11]. Among expatriate workers in Zambia and Ghana, only 44% and 11%, respectively, used chemoprophylaxis [12,13]. Similarly, business travelers with good understanding of malaria risk also failed to use appropriate personal protection measures when trip duration increased [14]. Reasons for noncompliance may include fear of long-term side effects, medication fatigue, adverse events, conflicting advice, perceived low risk of malaria, and complacency. Awareness of these issues will allow travel medicine practitioners to better prepare at-risk expatriate travelers [15,16].

GI infections, including acute diarrhea, cause significant morbidity among returned travelers globally [1,5,17]. Our study found that expatriates overall had lower PM for acute diarrhea and bacterial GI disease than non-expatriates. Returned expatriates had higher PM for other GI disorders, so GI illness remains an important source of morbidity for post-travel assessment. One possible explanation is that with longer exposure, partial immunity to acute bacterial pathogens may develop over time. This effect was seen in expatriates living in a highly endemic environment in Nepal, where a decreasing linear relationship was found between the odds ratio for diarrhea and duration of residence in that country [18]. This could also be due to changes in behavior by expatriates over the course of time. Another explanation would be the limitation of our proportionate morbidity methodology; expatriates could have higher rates and risks for these conditions but lower PM due to yet higher risks for other conditions.

Few studies distinguish business expatriates as a subgroup, distinct from missionaries, volunteers or aid workers. We found business expatriates had higher PM for vector-borne and vaccine-preventable infections, probably due to the broader range of occupations and countries of origin represented in our dataset. In our dataset, a lower proportion of business expatriates sought pre-travel consultation compared with missionary expatriates (33% vs. 67%). One implication of these findings is the importance of trying to reach business expatriates with pre-travel consultations, and focusing the encounter on preventing malaria, dengue and other vector-borne infections, and vaccine-preventable diseases such as hepatitis A. HIV infection and animal bites may also represent important but sometimes neglected topics for preventive advice and vaccination among business expatriates. However, these findings are based on low numbers, so the data should be interpreted with caution.

Volunteer expatriates were observed to have higher PM for parasitic infections, possibly reflecting greater rural exposure. Guidance for these travelers may need to focus on preventing parasites transmitted via mosquitoes (filariasis), ingestion (amebiasis), and environmental contact (schistosomiasis, strongyloidiasis), as well as anxiety/stress issues. Compared with non-expatriates, stressors experienced by expatriates may include a greater need to adapt to the culture, language, or infrastructure of the host country, loss of usual social supports, or work problems. Estimates of mental health problems range from 4% of health events in Peace Corps volunteers [19] to 10% in a study of British missionaries in 27 countries [8], with depression reported as the most common reason for psychiatric assessment [20]. Volunteer expatriates, such as aid workers or missionaries, may experience more psychological stressors. A retrospective study of 1250 returned Red Cross expatriates reported that 40% found their mission more stressful than expected, and 16% were exposed to at least one act of violence [21].

Although we excluded patients seen only for screening visits, we observed higher PM for latent TB among expatriates than non-expatriates, even after adjustment for age, sex, travel reason, travel duration, and region of exposure. Some of this difference may be due to screening protocols in different organizations. The higher PM for active TB among expatriates suggests these travelers may indeed have more exposure to TB. This finding is consistent with other studies which showed US Peace Corps volunteers with purified protein derivative (PPD) conversion rates of 1.3 per 1,000 volunteer-months [22], probably reflecting a true increased exposure to TB in higher-incidence regions.

Several negative findings in this analysis are worth noting. Enteric fever (typhoid/paratyphoid) is the most common bacteremic disease affecting travelers to the tropics [23], but in our study, we did not find any significantly different PM for enteric fever among expatriates as compared to non-expatriates. We found no cases of Japanese encephalitis, cholera, or meningococcal disease among the 14,793 ill returned expatriates and non-expatriates. Because these are rare diseases, our analysis may lack sufficient power to detect very low-incidence illnesses, which is a potential limitation of this study.

Our analysis using GeoSentinel data has several other important general limitations. First, the data do not represent a comprehensive epidemiologic analysis, with uniform and comprehensive sampling of all travel-related illnesses. Therefore, more severe or complex diseases may be overrepresented because of referrals to specialized GeoSentinel clinics, along with an underrepresentation of mild or self-limited conditions normally seen at nonspecialized primary care practices. Furthermore, diseases with short incubation periods with onset during the trip abroad (such as acute diarrhea) would also be underrepresented because the sample contains data only for post-travel patients. Second, because a comparison group of healthy travelers is unavailable, rates and risks could not be calculated for travel-related illness in expatriates and non-expatriates. Our odds ratio results should be interpreted accordingly. Last, high proportions of missing values were found for region of exposure and trip duration (9% and 10%, respectively); however, comparisons of missing to non-missing produced no statistical differences for demographic variables such as sex and age; these comparisons were included in the adjusted multivariate logistic regression.

After adjusting for demographics, travel reason, trip duration or region of exposure, our results indicate that expatriate travel did not have increased odds for some of the illnesses we are concerned about such as malaria. Malaria is seen commonly among expatriates but this may be due to longer duration of travel or region of exposure rather than the expatriate nature of travel. However, expatriates seen after travel do have increased adjusted odds for GI diseases other than acute diarrhea, and latent TB infection. The reliance of expatriates on local infrastructure, broader contact with endemic populations or behavior adaptations may increase expatriate exposure to these diseases. Understanding these results as well as the limitations of our data will further elucidate the role of expatriate travel and the factors that impact clinical outcomes in expatriates returning with symptomatic illness. We hope these insights will serve to inform the practice of clinicians and organizations serving expatriates, both in pre-travel preparation and post-travel consultations.

## Competing interests

PLL, PH, SM, PP, DH, PS, LL, XMD, DOF: None

LHC has received research funding from Xcellerex Inc and honoraria from Thompson Media LLC for serving on the editorial board of Travel Medicine Advisor. AWS has received speaker's honoraria from GSK, Sanofi Pasteur and Novartis, and serves on the Novartis Advisory Board for Travel Vaccines.

## Authors’ contributions

PLL conceived of the study, participated in study design, data analysis and interpretation, and drafted the manuscript. PH participated in study design, statistical analysis, and drafted the manuscript. LHC participated in study design, data interpretation, literature review and helped draft the manuscript. SM, PP, DH, PS, participated in data interpretation, literature review and helped draft the manuscript. LL, AWS participated in literature and manuscript review. XMD participated in study design and data analysis. DOF participated in data interpretation, and manuscript review. All authors read and approved the final manuscript.

## Disclaimer

The findings and conclusions in this report are those of these authors and do not necessarily represent the views of the Centers for Disease Control and Prevention.

## Pre-publication history

The pre-publication history for this paper can be accessed here:

http://www.biomedcentral.com/1471-2334/12/386/prepub

## References

[B1] Patel D Easmon C Seed P Dow C Snashall D Morbidity in expatriates – a prospective cohort study Occup Med 2006 56 345 352 10.1093/occmed/kql026 16717049

[B2] Dumont J Lemaitre G Counting immigrants and expatriates in OECD countries: A new perspective. OECD Social, employment and migration working papers No.25 (UN/POP/PD/2005/09) http://www.un.org/esa/population/migration/turin/Symposium_Turin_files/P09_Dumont&Lemaitre.pdf

[B3] Newson-Smith MS Importing health conditions of expatriate workers into the United Arab Emirates Asia Pac J Public Health 2010 22 25S 30S 10.1177/1010539510373021 20566530

[B4] Foyle MF Beer MD Watson JP Expatriate mental health Acta Psychiatr Scan 1998 97 278 283 10.1111/j.1600-0447.1998.tb10000.x 9570488

[B5] Freedman DO Weld LH Kozarsky PE Fisk T Robins R Spectrum of disease and relation to place of exposure among ill returned travelers N Engl J Med 2006 354 119 130 10.1056/NEJMoa051331 16407507

[B6] Chen LH Wilson ME Davis X Loutan L Schwartz E Illness in long-term travelers visiting GeoSentinel clinics Emerg Inf Dis 2009 15 11 1773 1782 10.3201/eid1511.090945 PMC2857257 19891865

[B7] Adera T Wolfe MS McGuire-Rugh K Calhoun N Marum L Risk factors for malaria among expatriates living in Kampala, Uganda: the need for adherence to chemoprophylactic regimens Am J Trop Med Hyg 1995 52 207 212 7694960 10.4269/ajtmh.1995.52.207

[B8] Peppiat R Byass P A survey of the health of British missionaries Br J Gen Pract 1991 41 159 162 1854537 PMC1371516

[B9] McLarty DG Webber RH Marjatta Jaatinen LM Kihamia CH Murru M Chemoprophylaxis of malaria in nonimmune residents in Dar-es-Salaam, Tanzania Lancet 1984 2 8404 656 659 6147692 10.1016/s0140-6736(84)91223-6

[B10] Leutscher PDC Bagley SW Health-related challenges in United States Peace Corps volunteers serving for two years in Madagascar J Travel Med 2003 10 263 7 14531978 10.2310/7060.2003.2690

[B11] Burdon J Use of malarial prophylaxis amongst a population of expatriate church workers in Northeast Zaire J Travel Med 1998 5 36 38 10.1111/j.1708-8305.1998.tb00455.x 9772315

[B12] Maarschalk T Ross MH de Frey AF Attitudes, knowledge, beliefs and practices regarding malaria prophylaxis in expatriate mine workers in the Zambian Copperbelt Occupational Health Southern Africa 2006 12 2 16 20

[B13] Hamer DH Ruffing R Callahan MV Lyons SH Abdullah AS Knowledge and use of measures to reduce health risks by corporate expatriate employees in western Ghana J Trav Med 2008 15 4 237 242 10.1111/j.1708-8305.2008.00214.x 18666923

[B14] Weber R Schlagenhauf P Amsler L Steffen R Knowledge, attitudes and practices of business travelers regarding malaria risk and prevention J Travel Med 2003 10 4 219 24 12946300 10.2310/7060.2003.40574

[B15] Toovey S Moerman F van Gompel A Special infectious disease risks of expatriates and long-term travelers in tropical countries. Part I: malaria J Trav Med 2007 14 1 42 49 10.1111/j.1708-8305.2006.00091.x 17241253

[B16] Chen LH Wilson ME Schlagenhauf P Prevention of malaria in long-term travelers JAMA 2006 296 18 2234 2244 10.1001/jama.296.18.2234 17090770

[B17] Greenwood Z Black J Weld L O’Brien D Leder K Gastrointestinal infections among international travelers globally J Trav Med 2008 15 4 221 228 10.1111/j.1708-8305.2008.00203.x 18666921

[B18] Hoge CW Shlim DR Echeverria P Rajah R Herrmann JE Epidemiology of diarrhea among expatriate residents living in a highly endemic environment JAMA 1996 275 7 533 538 10.1001/jama.1996.03530310039030 8606474

[B19] Keystone J Kozarsky P Freedman D Nothdurft H Connor B Travel Medicine 2004 Elsevier

[B20] Dally P Psychiatric illness in expatriates J Roy Coll Physicians Lond 1985 19 103 104 3999050 PMC5371014

[B21] Dahlgren AL Deroo L Avril J Bise G Loutan L Health risks and risk-taking behaviors among International Committee of the Red Cross (ICRC) expatriates returning from humanitarian missions J Trav Med 2009 16 382 390 10.1111/j.1708-8305.2009.00350.x 19930377

[B22] Jung P Banks RH Tuberculosis risk in US Peace Corps Volunteers, 1996–2005 J Trav Med 2008 15 2 87 94 10.1111/j.1708-8305.2008.00184.x 18346241

[B23] Paz A Cohen E Odeh M Potasman I Typhoid fever in travelers: time for reassessment Eur J Intern Med 2007 18 2 150 151 10.1016/j.ejim.2006.09.016 17338970

